# Emotional and Cognitive Responses and Behavioral Coping of Chinese Medical Workers and General Population during the Pandemic of COVID-19

**DOI:** 10.3390/ijerph17176198

**Published:** 2020-08-26

**Authors:** Zemin Cai, Shukai Zheng, Yanhong Huang, Xuanzhi Zhang, Zhaolong Qiu, Anyan Huang, Kusheng Wu

**Affiliations:** 1 Department of Preventive Medicine, Shantou University Medical College, Shantou 515041, China; 19zmcai@stu.edu.cn (Z.C.); 15skzheng@stu.edu.cn (S.Z.); 18zlqiu@stu.edu.cn (Z.Q.); 2 Mental Health Center, Shantou University, Shantou 515065, China; 12yhhuang@stu.edu.cn (Y.H.); xzzhang@stu.edu.cn (X.Z.); 19ayhuang@stu.edu.cn (A.H.)

**Keywords:** Corona Virus Disease 2019, emotion, cognition, behavioral coping

## Abstract

Background: The outbreak of Corona Virus Disease 2019 (COVID-19) might affect the psychological health of population, especially medical workers. We aimed to investigate the impact of the COVID-19 pandemic on emotional and cognitive responses and behavioral coping among Chinese residents. Methods: An online investigation was run from 5 February to 25 February 2020, which recruited a total of 616 Chinese residents. Self-designed questionnaires were used to collect demographic information, epidemic knowledge and prevention of COVID-19 and characteristics of medical workers. The emotional and cognitive responses were assessed via the Symptom Check List-30 (SCL-30) and Yale–Brown Obsessive Compulsive Scale (Y-BOCS). Behavioral coping was assessed via Simplified Coping Style Questionnaire (SCSQ). Results: In total, 131 (21.3%) medical workers and 485 (78.7%) members of the general public completed the structured online survey. The structural equation models showed that emotional response interacted with cognitive response, and both emotional response and cognitive response affected the behavioral coping. Multivariate regression showed that positive coping enhanced emotional and cognitive responses, while negative coping reduced emotional and cognitive responses. The emotional response (depression, anxiety and photic anxiety) scores of the participants were higher than the norm (all *p* < 0.001); in particular, the panic scores of members of the general public were higher than those of medical workers (*p* < 0.05), as well as the cognitive response (paranoia and compulsion). Both positive and negative coping scores of the participants were lower than the norm (*p* < 0.001), and the general public had higher negative coping than medical workers (*p* < 0.05). Conclusion: During the preliminary stage of COVID-19, our study confirmed the significance of emotional and cognitive responses, which were associated with behavioral coping and significantly influenced the medical workers and the general public’s cognition and level of public health emergency preparedness. These results emphasize the importance of psychological health at times of widespread crisis.

## 1. Introduction

In December 2019, Corona Virus Disease 2019 (COVID-19) was first reported and became an outbreak in Wuhan, the capital city of Hubei Province, China [1,2]. The disease, which was caused by coronavirus SARS-CoV-2, rapidly spread throughout China and become a global health emergency [3]. On March 11, 2020, the World Health Organization (WHO) declared COVID-19 a pandemic.

The psychological health of people, especially medical workers, has been greatly challenged during the immediate wake of the viral pandemic. People have high levels of stress due to there being no firm estimate of how long the pandemic will last and how long their lives will be disrupted or whether they will be infected [4]. Moreover, the lack of sufficient knowledge about COVID-19, various channels of opaque information, various rumors, fear of infection, and prolonged isolation and confinement, might seriously affect their psychological health. Being isolated, working in high-risk positions, and having contact with infected people are common causes of psychological burden among medical workers [5,6].

According to psychology, public health emergencies are a negative pressure source, with characteristics of suddenness, menace, extensiveness and infectivity, and people’s life and health have been greatly threatened [7,8]. Public health emergencies, such as COVID-19, can cause depression, anxiety, photic anxiety and other psychological responses, and lead to the emergence of stress disorder, post-traumatic stress disorder and other psychological disorders [4,9]. These psychological barriers of negative emotion can occur even in people not at high risk of getting sick, in the face of a virus with which the general public may be unfamiliar [10].

A precondition for carrying out effective emergency intervention is to evaluate and analyze the psychological state of the people affected by the emergency and to understand the characteristics of the emotional and cognitive responses of different groups in time, so as to take efficient and comprehensive actions in a timely fashion to protect the psychological health of people, especially medical workers [9].

Therefore, with reference to the ‘Corona Virus Disease 2019 Guidelines for Public Psychological Self-help and Counseling’ [11] and the ‘Compilation of Psychological Responses Questionnaire to Public Health Emergency’ [12], this study was mainly carried out in terms of three aspects—emotional response, cognitive response, and behavioral coping—with the aim of evaluating the psychological health status and behavioral coping of medical workers and the general public under the COVID-19 pandemic, and exploring the impact of public health emergencies on psychological health, so as to provide a basis from which relevant departments can carry out precise prevention and control strategies for the public. A structural equation model (SEM) was established to verify the hypothetical associations among emotional and cognitive responses and behavioral coping. The hypotheses were that emotional response interacted with cognitive response, and the psychological dimensions of emotional and cognitive responses affected behavioral coping.

## 2. Materials and Methods 

### 2.1. Participants

This is a cross-sectional study performed via an online investigation run from February 5 to February 25, 2020. We recruited Chinese residents faced with the COVID-19 pandemic, including medical workers and members of the general public, to participate in this investigation. The research project was approved by the human ethics committee of the Mental Health Center of Shantou University in accordance with the Declaration of Helsinki. All participants provided written informed consent and received a small gift as compensation for their involvement.

Data were collected through Wenjuanxing (http://www.wjx.cn, Changsha Ranxing Information Technology Co., LTD, Changsha, China) with an anonymous, self-rated questionnaire that was distributed to network platforms. All participants were informed of the contents of the study and advised of their privacy and confidentiality commitments when volunteering. Each IP address was accessed to respond once. The answering time of each questionnaire was automatically monitored in the network background, and to guarantee the validity and feasibility of the questionnaire, questionnaires answered in less than 100 s were regarded as invalid.

### 2.2. Questionnaires

The questionnaire includes two parts: basic demographic information and psychological health assessment, including emotional response, cognitive response and behavioral coping. 

#### 2.2.1. Demographic Data

Demographic data included basic demographic characteristics, acquisition and cognition of preventive knowledge, factors affecting mood, basic information and pressure source of medical workers.

#### 2.2.2. Psychological Health Assessment

Three scales were used to assess the psychological health status and coping styles of participants. The Symptom Checklist-30 scale (SCL-30) [13] and the Yale–Brown Obsessive Compulsive Scale (Y-BOCS) [14] were used to evaluate the emotional response and cognitive response. The Simplified Coping Style Questionnaire (SCSQ) [15] was used to evaluate behavioral coping.

SCL-90-R [16], which was developed in its initial version by Derogatis LR [17] and will be revised soon, includes a wide range of psychiatric symptomatology. This survey addresses emotional response (depression, anxiety and photic anxiety) and cognitive response (paranoia and compulsion). Depression, anxiety, photic anxiety and paranoia were selected from SCL-90-R to constitute SCL-30. The SCL-30 scale, a 30-item self-reported scale with items rated on a 5-point Likert scale, was used to select the dimensions of evaluating emotion during a public health emergency, including depression, anxiety, photic anxiety, paranoia, with the scores categorized as follows: no (0), mild (1), moderate (2), severe (3), or pretty severe (4). 

The Y-BOCS includes two subscales, corresponding to obsessive thinking and compulsive behavior, respectively. The scale covers the core attributes of obsessive symptoms and can comprehensively evaluate the severity of obsessive symptoms. The severity was assessed via symptomatic distress, frequency, conflict, and self-resistance. The subscales were revised by psychologist on the basis of pandemic of COVID-19, with the scores categorized as follows: complete control (0), a majority of control (1), moderate control (2), poor control (3), or lost control (4). A total score of 6–15 indicates mild compulsion, 16–25 indicates moderate compulsion, and more than 25 indicates severe compulsion.

The SCSQ was synthesized by coping styles cognitive theories and combined with the Chinese characteristics. SCSQ included 20 evaluation points, and was used to evaluate population positive coping and negative coping during an emergency, with the scores categorized as follows: not adopt (0), occasionally (1), sometimes (2), usually (3). The positive coping dimension consisted of items 1–12, which mainly reflect the characteristics of positive coping. The negative coping dimension consisted of items 13–20, which mainly reflect the characteristics of negative coping. 

### 2.3. Statistical Analysis

Statistical analyses were performed with IBM SPSS Statistics 26.0 (IBM Corp., Armonk, NY, USA) and GraphPad prism 8 (GraphPad Software Inc., San Diego, CA, USA). Descriptive analyses were used for demographic data. The results were expressed as the percentage value for categorical data and mean ± standard deviation for continuous data. Chi-square tests were used to compare the data for different categorical variables. All tests were two-sided and *p* < 0.05 was considered statistically significant. Reliability factor analysis was performed using IBM SPSS Statistic 26.0 for the reliability of the scale, and confirmatory factor analysis was performed with Amos22.0 (IBM Corp., Armonk, NY, USA) for the validity of the scale. A structural equation model (SEM) was constructed via Amos22.0 to explore the relationship among emotional response, cognitive response and behavioral coping. Multivariate stepwise regression analyses were performed using stepwise variable selection to further analyze the association among emotional and cognitive responses and behavioral coping.

## 3. Results

### 3.1. Demographic Characteristics of the Participants

A total of 616 participants, including 131 (21.3%) medical workers and 485 (78.7%) members of the general public, completed the online survey. Table 1 presents the demographic characteristics of the participants. More females (63.8%) participated in this survey, and 57.8% of the participants were aged from 19 to 35 years. The participants had relatively high educational levels, with more than half of participants (71.6%) having an educational level of undergraduate or higher. The majority of participants (97.1%) did not have a contact history of COVID-19.

### 3.2. Acquisition and Cognition of Preventive Knowledge of COVID-19

The main methods of acquisition of information among medical workers and the general public were official news and broadcasts, chat software, and timely messages from the APP, with statistical significance between the two groups (*p* = 0.005). Additionally, awareness of preventive measures was statistically significant (*p* = 0.029) among the two groups, such as wearing a mask, avoiding gathering together, washing hands, disinfecting furniture, and maintaining healthy habits. Factors affecting mood were mainly limited to going outside, going to work early, and the impact on the original schedule, with statistical significance (*p* < 0.001) being exhibited between the two groups (Table 2).

### 3.3. General Characteristics of Medical Workers and Their Pressure Sources

**Figure 1:**
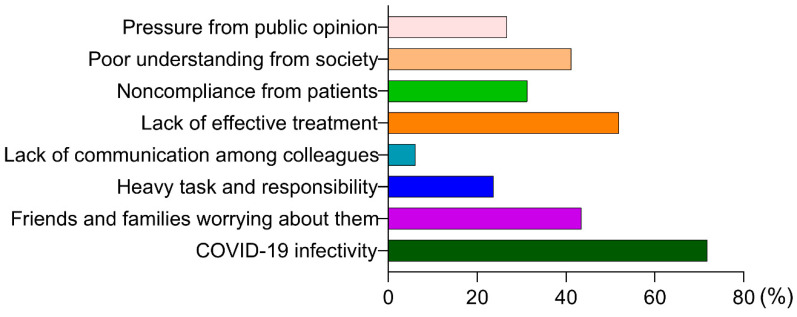
The pressure sources of medical workers.

A total of 131 medical workers participated in the survey, including 46 (35.1%) clinical doctors, 20 (15.3%) public health physicians and 30 (22.9%) nurses. 36.6% of the medical workers worked in the designated hospital for COVID-19, and 40.5% worked in the general hospital. A total of 46.6% worked in clinical departments, 8.4% worked in epidemic prevention or infection control and 15.3% worked in the auxiliary department (Table 3). Their top three pressure sources were, in turn, the highly contagious nature of COVID-19, the lack of effective treatment, and their relatives and friends worrying about them (Figure 1).

### 3.4. Association among Emotional and Cognitive Responses and Behavioral Coping

#### 3.4.1. Reliability and Validity of Emotional and Cognitive Response Subscales

**Figure 2:**
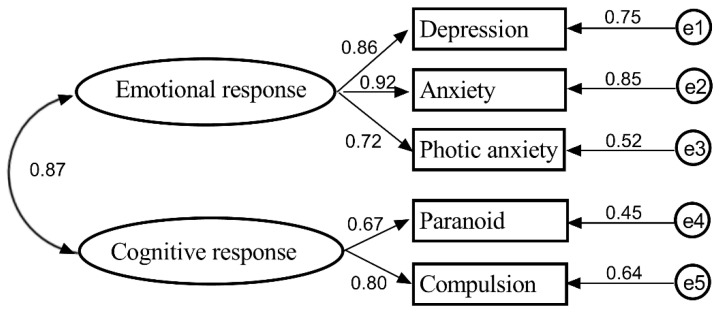
Confirmatory factor analysis of emotional and cognitive response scales.

IBM SPSS Statistic 26.0 was used to analyze the reliability of the emotional and cognitive responses subscale. The Cronbach’s α coefficient of the emotional response subscale was 0.813, the Cronbach’s α coefficient of the cognitive response subscale was 0.820, and the Cronbach’s α coefficient of the coping style questionnaire was 0.871. Amos 22.0 was used to analyze the validity α of the emotional response subscale and the cognitive response subscale. Parameter estimation was computed by the maximum likelihood estimate. In addition, standard regression weights of the emotional response subscale and cognitive response subscale are shown in Figure 2. General standard regression weight is required > 0.7. The Cronbach’s α and standard regression weight describe the explanatory power of latent variables for the measured variables.

#### 3.4.2. SEM of Emotional and Cognitive Responses and Behavioral Coping

**Figure 3:**
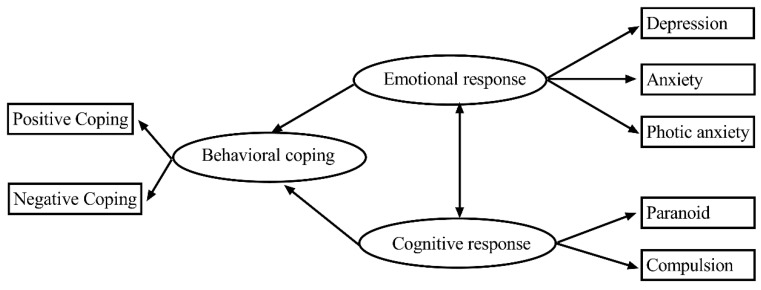
SEM of emotional and cognitive responses and behavioral coping.

We established an SEM of the associations among emotional response, cognitive response and behavioral coping. Firstly, emotional response as a respective factor for psychological health, including depression, anxiety and photic anxiety, was analyzed in the previous step. Secondly, cognitive response consisted of paranoia and obsessive compulsion. The third area was the simplified coping style of the population regarding whether their coping was positive or negative during the pandemic (Figure 3). The Chi-square test of model fit yielded a value (CMIN) of 116.74, with degrees of freedom = 18, *p* < 0.001, RMSEA = 0.022, CFI = 0.947, IFI= 0.947 and TLI = 0.907, indicating a good fit. The results showed that emotional response interacted with cognitive response. In addition, the psychological dimensions of emotional response and cognitive response affected behavioral coping. The results are shown in Figure 3 and Table 4.

#### 3.4.3. Multivariate Stepwise Regression among Emotional and Cognitive Responses and Behavioral Coping

Multivariate stepwise regression was used to analyze the association among emotional and cognitive responses and behavioral coping. The emotional and cognitive response scales’ total scores were regarded as dependent variables, and behavioral coping, including positive coping and negative coping, was regarded as an independent variable. The results showed that both positive coping (β= −0.21) and negative coping (β = 0.53) were significantly associated with emotional and cognitive responses (*R*^2^ = 0.177, *F* = 111.34, *p* < 0.001), and positive coping enhanced emotional and cognitive responses, while negative coping reduced emotional and cognitive responses (Table 5).

#### 3.4.4. Comparison of the Scales’ Scores between Medical Workers and the General Public

In emotional response, the depression, anxiety and photic anxiety scores among medical workers and the general public were higher than the norm (all *p* < 0.001), and the photic anxiety score of the general public was higher than that of medical workers (*p* < 0.05). As to cognitive response, the paranoia score among medical workers and the general public was higher than the norm (*p* < 0.05), and the compulsion sore of the general public was higher than that of medical workers (*p* < 0.05) (Table 6). Both positive coping and negative coping scores among medical workers and the general public were lower than the norm (both *p* < 0.001), while the negative coping sores of the general public were higher than those of medical workers (*p* < 0.05) (Table 7).

## 4. Discussion

This is the first investigation of emotional and cognitive responses and behavioral coping in the wake of the coronavirus epidemic in China that explores psychological health in cases of public health emergency. To conduct a comprehensive analysis, we used multiple scales to evaluate the emotional and cognitive responses and behavioral coping of the Chinese population, especially medical workers.

When cities are struck by various disasters, the characteristics of psychological health problems that arise can differ in different periods [18,19]. After an emergency, people suffering from impacts on their psychological health often outnumber people who are physically injured, and psychological health impacts may last longer [20]. The viral pandemic, as a huge negative pressure source, poses great challenges to the psychological health of people, especially medical workers [21]. Although the majority of Chinese residents did not have a contact history of COVID-19 in this survey, complex emotions can occur even in people not at high risk of getting sick, in the face of a virus with which the general public may be unfamiliar [22]. 

People received information via various channels. Distinguishing real news from rumors undoubtedly increases the psychological burden of the public. Medical workers tend to be able to distinguish real news and rumors because of their expert knowledge. The Preventive Guidelines for People at Different Risks of SARS-CoV-2 Infection [23] were promulgated on time, and the public were generally aware of the transmission of COVID-19, but were still puzzled as to how to prevent it. Thus, acquisition and awareness of preventive knowledge among medical workers and the general public were of statistical significance, probably because medical workers have professional knowledge and occupational skills [24,25]. 

In terms of the SEM in this study, emotional response interacted with cognitive response, and the psychological dimensions of emotional response and cognitive response affected behavioral coping. The results of multivariate stepwise regression showed that positive coping may enhance psychological health, while negative coping may reduce psychological health. Both positive and negative coping had significant predictive power for emotional and cognitive responses. The study [5] confirmed that people mainly transformed their assessment of stress events and adopted corresponding coping strategies to adjust their emotional and cognitive responses. The relationship between individual coping styles and psychosomatic health has become an important content of psychological research [26,27,28]. The circumstances of pressure sources are evaluated to judge whether it burden or exceed the individual coping skills or not. This study found that mild psychological health disturbances accounted for a large proportion. People with mild psychological disturbances may be more likely to adopt coping styles and learn the necessary skills, so as to adapt in productive ways and cope with different challenges. Previous retrospective studies [6,29,30] have shown that the coping ways and necessary skills were protective for psychological health.

The results showed that the emotional and cognitive response scores increased, while behavioral coping scores decreased in both the general public and among medical workers. In addition, the scores of each psychological dimension and the coping dimension of the general public were higher than those of medical workers, among which the differences in compulsion and negative coping were statistically significant. During the pandemic, both medical workers and the general public’s psychological health were affected, and medical workers have a better emotional and cognitive responses and coping styles to public health emergencies than the general public. Medical workers with professional knowledge in relative exposure patterns and transmission of various infectious diseases could acquire some degree of comfort and control over their situations [24].

People have high levels of stress due to there being no definite estimate of how the long pandemic will last and how long our lives will be disrupted, or whether we will be infected. Additionally, long-term limitations of going out, impact on original schedule, lack of social interaction and mixed information have an influence on people’s psychological health [19,31]. In this study, the causes of psychological burden on medical workers were the infectivity of COVID-19, the lack of effective treatment, the initially insufficient understanding of the virus, and poor support from society and patients. For medical workers, their life status of daily fighting against COVID-19 shows that they have to be able to cope with psychological pressure and are at risk of allostatic load [32]. In pandemic situations, such exposure is known to be mentally injurious [33,34]. Not only does the direct exposure of the work circumstances affect the psychological health of medical workers, but the infection of close relatives generated psychological trauma or fear when the public health emergency hit [5]. 

Studies [35,36,37] have shown that there is a positive correlation between psychological health and occupational stress. Excessive occupational stress will aggravate anxiety, panic and other adverse psychological emotions of medical workers, as well as somatic symptoms such as insomnia and digestive tract abnormalities, causing negative effects on their work. In the meantime, these impacts reinforce occupational stress in turn. Subsequently, with training on the Novel Coronavirus Infection Pneumonia Diagnosis and Treatment Plan for all medical workers [38], with continuously updated guidelines on how to deal with COVID-19 patients [39], with rest in shifts for medical workers, and with rapid supply of medical protective items, the stress of medical workers has been relieved to some degree and supporting their perseverance. Therefore, relevant departments and institutions should carry out targeted psychological guidance and intervention in the population, especially for medical workers during the pandemic, as well as for reconstruction after the pandemic. Medical workers should be given more social support and understanding, so as to protect the solid “defense line” during infectious disease outbreaks.

Limitations and strengths. The present study has several limitations. First, this study was based on an online survey. The use of clinical interviews is encouraged to draw a more comprehensive assessment of the problem in future studies. Second, this study just reflects people’s psychological health at a particular phase of the COVID-19 pandemic, and a longitudinal approach might help research the development of allostatic overload and changes in post-traumatic psychological health. Despite these limitations, multiple dimensions were considered in this study to analyze people’s psychological characteristics during the pandemic. In addition, an SEM was established to evaluate the associations among the emotional response, cognitive response and behavioral coping of Chinese residents. In the future, somatic symptoms, such as insomnia, nausea, vomiting, anorexia, and frequent urination, could also appear during public health emergencies in addition to emotional responses and cognitive responses. Somatic symptoms can be considered as another subscale to interact with emotional response and cognitive response, and the SEM can thus be further refined to explore the influence on the pandemic of COVID-19. Research in post-traumatic psychological health, dynamic observation and psychological intervention should be performed to obtain more epidemiological data and more specific clues for the intervention of psychological health.

## 5. Conclusions

During the preliminary stage of COVID-19, our study confirmed the significance of emotional response and cognitive response, which are associated with behavioral coping and significantly influenced the medical workers and the general public’s cognition and level of public health emergency preparedness. It is necessary to recognize psychological health needs as an important component of response to sudden city-scale crisis scenarios.

## Figures and Tables

**Table 1:** Demographic characteristics of the participants.

Variables	Number (*n* = 616)	Percentage (%)
Gender		
Male	223	36.2
Female	393	63.8
Age (years)		
0–18	55	8.8
19–35	356	57.8
36–59	188	30.5
≥ 60	17	2.8
Educational level		
Secondary school or less	57	9.2
Junior college	118	19.1
Undergraduate	280	45.5
Postgraduate or more	161	26.1
Occupation		
Health workers	131	21.3
Educational and cultural workers	59	9.6
Students with medical background	90	14.6
Students without medical background	66	10.7
Civil servant/Career preparation	64	10.4
Company employee	92	14.9
Service industry	17	2.8
Self-employment venture	19	3.1
Worker/Farmer	13	2.1
Full-time housewife	6	1.0
Retired	14	2.3
Others	45	7.3
COVID-19 contact history		
No	598	97.1
Contact history of epidemic focus	4	0.6
Suspected or confirmed COVID-19 patients living around	14	2.3

**Table 2:** Acquisition and awareness of preventive knowledge between medical workers and the general public.

Variables	Medical Workers (*n* = 131)	Public (*n* = 485)	*p*-Value
Main access of information			0.005
Official news and broadcasts	114 (87.0)	412(84.9)	
Short message service	58 (44.3)	252(52.0)	
Chat software	68 (51.9)	325(67.0)	
Timely message from APP	85 (64.9)	347(71.5)	
Video Clips from APP	19 (14.5)	114(23.5)	
Internet searching	59 (45.0)	217(44.7)	
Others	7 (5.3)	23(4.7)	
Awareness of COVID-19 transmission			0.35
Droplet transmission	131(100.0)	482(99.4)	
Contact transmission	123(93.9)	428(88.2)	
Fecal–oral transmission	99(75.6)	373(76.9)	
Household articles transmission	85(64.9)	337(69.5)	
Others	8(6.1)	35(7.2)	
Awareness of preventive measures			0.029
Wear a mask	129(98.5)	484(99.8)	
Avoid gathering together	126(96.2)	481(99.2)	
Wash hands and disinfect furniture	128(97.7)	477(98.4)	
Exercise	109(83.2)	427(88.0)	
Raise the room temperature	19(14.5)	84(17.3)	
Healthy living habits	121(92.4)	432(89.1)	
Vinegar vapors	8(6.1)	45(9.3)	
Factors affecting mood			<0.001
Limited going outside	55(42.0)	361(74.4)	
Going to work early	42(32.1)	59(12.2)	
Impact on the original schedule	60(45.8)	235(48.5)	
Poor preventive awareness of families	25(19.1)	110(22.7)	
Unable to reunite with families	43(32.8)	80(16.5)	
Information explosion and rumors	67(51.1)	237(48.9)	
Suspected cases around residence	35(26.7)	97(20.0)	
Others	8(6.1)	29(6.0)	

**Table 3:** General characteristics of participating medical workers (*n* = 131).

Characteristics	Number	Percentage (%)
Work units		
Designated hospital for COVID-19 patients	48	36.6
General hospital	53	40.5
Disease prevention and control department	7	5.3
Community health service center	10	7.6
Others	13	10.0
Concrete post		
Clinician	46	35.1
Public health physician	20	15.3
Nurse	30	22.9
Administrative staff	12	9.2
Others	23	17.6
Work department		
Clinical department	61	46.6
Radiology department	4	3.1
Clinical Test Lab	5	3.8
Epidemic prevention or infection control department	11	8.4
Auxiliary department	20	15.3
Others	30	22.9
Seniority		
<5 years	53	40.5
5–10 years	34	26.0
10–20 years	29	22.1
20 years	15	11.5

**Table 4:** Direct and indirect effects in SEM.

Direct or Indirect Effects Pathway	Estimate	Standard Error	C.R.	*p*-Value
Depression←Emotion	13.723	1.410	12.033	<0.001
Anxiety←Emotion	4.349	0.532	8.168	<0.001
Photic anxiety←Emotion	8.3885	0.530	15.817	<0.001
Paranoia←Cognition	4.799	0.286	16.787	<0.001
Compulsion←Cognition	23.275	1.448	16.077	<0.001
Positive←Behavior	0.499	0.043	11.721	<0.001
Negative←Behavior	−0.123	0.073	−1.678	0.093
Behavior←Emotion	35.019	2.793	12.537	<0.001
Behavior←Cognition	2.779	0.345	8.067	<0.001

**Table 5:** Multivariate stepwise regression among emotional and cognitive responses and behavioral coping.

Model	B	Beta	t	*p*-Value	95%CI
Constant	11.68	—	7.77	<0.001	(8.73, 14.64)
Positive	−4.85	−0.21	−4.15	<0.001	(−7.15, −2.55)
Negative	15.05	0.53	10.71	<0.001	(12.29, 17.81)

*F* = 111.34, *p* < 0.001, *R*^2^ = 0.177.

**Table 6:** Comparison of emotional and cognitive responses sores between participants and norm.

Response	Dimensions	Norm	Medical Workers	Public	*F*	*p*-Value
Emotion	Depression	1.50 ± 0.59	4.42 ± 6.16	4.89 ± 7.20	68.22	<0.001
	Anxiety	1.39 ± 0.43	2.85 ± 4.96	2.91 ± 5.03	27.65	<0.001
	Photic anxiety	1.23 ± 0.41	2.69 ± 4.01	3.35 ± 3.98 *	78.02	<0.001
Cognition	Paranoia	1.43 ± 0.57	1.56 ± 2.48	1.79 ± 2.83	4.44	0.01
	Compulsion	6~10 (Mild)	6.22 ± 6.25	7.36 ± 7.01 *	54.40	<0.001

*F*-value is statistic of ANOVA test among the norm, medical workers and general public groups; * Comparison between medical workers and the general public, *p* < 0.05.

**Table 7:** Comparison of behavioral coping sores between participants and norm.

Behavioral Coping	Norm	Medical Workers	Public	*F*	*p*-Value
Positive	1.78 ± 0.52	1.35 ± 0.91	1.48 ± 0.88	32.97	<0.001
Negative	1.59 ± 0.66	0.78 ± 0.73	1.06 ± 0.73 *	118.89	<0.001

*F*-value is statistic of ANOVA test among the norm, medical workers and general public groups; * Comparison between medical workers and the general public, *p* < 0.05.
